# Pragmatic evaluation of methods for retrieving unpublished information on comparator interventions in a systematic review of smoking cessation trials

**DOI:** 10.1080/08870446.2022.2081688

**Published:** 2022-07-23

**Authors:** Neža Javornik, Daniel J. H. Powell, Maarten C. Eisma, Marie Johnston, Marion K. Campbell, Jamie Hartmann-Boyce, Susan Michie, Robert West, Nicola Black, Marijn de Bruin

**Affiliations:** a Health Psychology Group, Institute of Applied Health Sciences, University of Aberdeen, Aberdeen, United Kingdom.;; b Rowett Institute, University of Aberdeen, Aberdeen, United Kingdom.;; c Department of Clinical Psychology and Experimental Psychopathology, University of Groningen, Groningen, the Netherlands.;; d Technology Addiction Team, Brain and Mind Centre, University of Sydney, Australia.;; e Health Services Research Unit, Institute of Applied Health Sciences, University of Aberdeen, Aberdeen, United Kingdom.;; f Nuffield Department of Primary Care Health Sciences and National Institute of Health Research Oxford Biomedical Research Centre, University of Oxford, Oxford, United Kingdom.;; g Centre for Behaviour Change, University College London, London, United Kingdom; h Department of Behavioural Science and Health, University College London, London, United Kingdom; i Radboud University Medical Center, Radboud Institute for Health Sciences, IQ Healthcare, Nijmegen, the Netherlands.

**Keywords:** reporting, comparator, control group, validity, systematic review, behaviour change techniques

## Abstract

**Objective:**

Reporting of the content and delivery characteristics of comparator interventions in published articles is often incomplete. This study examines the feasibility and validity of two methods for collecting additional information on comparator interventions from trial authors.

**Methods & Measures:**

In a systematic review of smoking cessation trials (IC-Smoke), all trial authors were asked to send unpublished comparator intervention materials and complete a specially-developed comparator intervention checklist. All published and additionally obtained information from authors were coded for behaviour change techniques (BCTs) and other characteristics (type of comparator, provider, provider training, delivery mode and treatment duration). To assess representativeness, we assessed the amount of additional information obtained from trial authors compared with the amount that was published. We examined known-group and convergent validity of comparator intervention data when using only published or also unpublished information.

**Results:**

Additional information were obtained from 91/136 (67%) of trial authors. Representativeness, known-group and convergent validity improved substantially based on the data collected by means of the comparator intervention checklist, but not by requesting authors to send any existing comparator materials.

**Conclusions:**

Requesting authors for unpublished comparator intervention data, using specially-developed checklists and unpublished materials, substantially improves the quality of data available for systematic reviews.

## Introduction

Comprehensive reporting of behavioural interventions is key to evidence synthesis and intervention replication and implementation (Chalmers & Glasziou, 2009; Boutron et al., 2008). Despite the development of multiple reporting guidelines such as CONSORT-SPI (Montgomery et al., 2018) or CONSORT Extension for Non-pharmacological trials (Boutron et al., 2008; 2017), intervention descriptions are often incomplete (Black et al., 2020; de Bruin et al., 2021; Hoffmann et al.,^2013^; Chalmers & Glasziou, 2009). Reviews suggest that the descriptions of interventions delivered to comparator interventions may be even poorer than of those delivered to experimental groups (Byrd-Bredbenner et al., 2017; Ayling et al., 2015; Dombrowski et al., 2012; de Bruin et al., 2010; de Bruin et al., 2009), particularly regarding the potential active components of comparator interventions; and have not improved with time. Yet, comprehensive descriptions of comparator interventions are important to the interpretation and comparison of intervention effects (de Bruin et al., 2009, 2010; Wagner & Kanouse, 2003). Several methods have been used in the context of systematic reviews to collect unpublished data on comparator interventions from trial authors; but an in-depth evaluation of their properties is lacking. Even if the comparator interventions were comprehensively reported from now on, the evidence of poor reporting of the comparators (Byrd-Bredbenner et al., 2017) means that the effects of comparators’ active content from the majority of already-published trials cannot be adequately interpreted or compared in evidence synthesis. The current study examined the properties of two methods for retrospectively obtaining unpublished information on comparator interventions from trial authors, as part of a larger systematic review of randomised controlled trials of smoking cessation (De Bruin et al., 2016; de Bruin et al., 2021; Black, Eisma, et al., 2020; Black, Johnston, et al., 2020).

### Methods for collecting unpublished data

By reviewing the literature, two common approaches that systematic reviewers used to collect privately-held information from study authors were identified. The first approach consists of contacting the study authors with a request for any privately-held materials describing the intervention delivered, such as leaflets, manuals, or protocols (e.g. Albarqouni et al., 2018). In a systematic review of non-pharmacological interventions, Hoffmann et al. (2013) asked the authors for privately-held materials of recently published trials (2009). That improved the overall percentage of well-reported trials (as defined in that study) from 39% to 59%, but it had a much more substantial effect on the most poorly reported trial characteristic, namely the presence of intervention materials (increased from 47% to 92%). Similarly, Albarqouni et al. (2018) contacted the authors in a systematic review of educational interventions, which improved the completeness of main reporting criteria in 20% of studies. Recently published papers (2010-2016) could be rated as complete in 41% after contacting study authors in comparison to 5% of older studies (<2005). After contacting study authors (response rate: 48%), missing intervention materials (again the most poorly reported trial characteristic) were recovered for 25% of studies. It seems that simply asking authors for unpublished materials is a promising method for recovering information on interventions. It is important to note that these figures relate to experimental interventions (not comparators) and that recovering information appears to be better for recently published trials. Lastly, these studies did not examine how informative responses were and only coded whether unpublished materials were obtained.

A second approach by de Bruin and colleagues (2009) was developed specifically for assessing the content of treatment-as-usual (TAU) comparators. They constructed a checklist of potential active ingredients of comparator interventions, identified by consulting experts, literature, and guidelines in their topic area; and then sent the checklist to study authors for completion. The checklist was expected to yield more information than asking authors to send unpublished materials describing TAU, since TAU is—anecdotally—rarely formally documented (hence, asking authors for materials provides limited responses). Authors might remember, however, the care delivered at their study site(s) during their trial and could potentially report on it even years later. De Bruin and colleagues found that 63% (18/29) of study authors felt sufficiently confident in their knowledge of TAU to complete the checklist. The checklist showed a high internal consistency (α = .91) and authors’ responses predicted differences in health behaviour and clinical outcomes in comparators, as well as in trial effect sizes (i.e. predictive validity; de Bruin et al., 2009; 2010). In another systematic review of 20 trials, Ayling and colleagues (2015) adapted the approach from de Bruin and colleagues (with several modifications in the items and response scales) and found similar response rates (85%) and good internal consistency (α = .78), but not predictive validity (i.e. no differences in clinical outcomes between TAU in different trials).

### What information to collect on comparator interventions?

When collecting or reporting the information on comparator interventions, the characteristics that need to be considered are similar to those of experimental interventions. Several useful frameworks and tools have been developed to facilitate the reporting of experimental interventions (Montgomery et al., 2018; Hoffmann et al., 2014 Kok et al., 2016; Michie et al., 2013; Boutron et al., 2008). For example, the development of the 12-item Template for Intervention Description and Replication (TIDieR) offers a structure for reporting the essential elements of interventions (e.g. mode of delivery, intervention provider). TIDieR can also be used to examine how well the published interventions have been reported (e.g. Hoffmann et al., 2014).

The TIDieR framework applies to the reporting of interventions, in general. For individual domains, such as behavioural interventions, there are tools that can help to further specify characteristics of interventions. For example, researchers conducting behavioural trials can report the potential active content of interventions and comparators (TIDieR item ‘What’) by using the 93-item Behaviour Change Technique (BCT) Taxonomy (v1) (Michie et al., 2013) or the Behaviour Change Methods (BCMs) taxonomy (Kok et al., 2016). In the present study, we used the TIDieR framework and the BCT taxonomy to characterise the information required from comparator intervention descriptions in systematic reviews.

### Exploring the benefits of obtaining unpublished data

For systematic reviewers to devote resources to contacting study authors for missing data on experimental or comparator interventions, it is important to know the benefits of doing so. In particular, the present study examines author response rates and various aspects regarding the validity of the data collected. Specifically, the present study examines the representativeness of information about comparator intervention content when obtained by different methods. Representativeness here refers to the degree to which the elements of the assessment instrument are ‘proportional to the facets of the targeted construct’ (Haynes et al., 1995, p. 239), i.e. if it adequately portrays all the domains of the target construct (Haynes et al., 1995). In addition to content validity, construct validity examines whether the assessed instrument (in our instance, data collection methods) is relevant to and measures the targeted construct (in our instance, active content) (Haynes et al., 1995). Such construct validity can have different sub-types. For this study, two were selected: known-groups validity and convergent validity. Known-groups validity is the extent to which ‘a method can discriminate between groups, known to differ on the variables of interest’ (Davidson, 2014). Convergent validity refers to the extent to which data retrieved with our method is associated with variables we expect to measure a similar construct (Chin et al., 2014). These types of validity can be assessed by examining the relationships between identified presumed active content and different comparator intervention descriptors known to be associated with the quantity of delivered active content, such as type of comparator intervention or type of comparator intervention provider (known-groups validity), or treatment time (convergent validity), across different data sources.

The aim of this study was to compare the feasibility and validity of two approaches to collecting missing comparator data from trial authors, namely through requesting existing materials describing comparator interventions or the completion of the comparator intervention checklist. The study set out to answer the following research questions:

Are authors more likely to respond to our information request by sending unpublished materials or by completing the checklist?

How much additional information to that already available in the published reports can be retrieved by asking authors to send unpublished materials and to complete a purpose-built comparator intervention checklist?

Does the content validity (representativeness) of data collection methods on comparator intervention descriptions improve when, in addition to published data, unpublished data are obtained from study authors?

Does the construct validity of obtaining information on comparator interventions’ active content improve when, in addition to published data, unpublished data are obtained from study authors? Do methods used to obtain additional information yield stronger associations:

- … between types of providers and the intended number of BCTs in comparator interventions, than observed when obtaining published data only? (known-groups validity)
- … between types of comparators and the intended number of BCTs in comparator interventions, than observed when obtaining published data only? (known-groups validity)
- … between the treatment time of the comparator intervention and the intended number of BCTs in comparator interventions, than observed when obtaining published data only? (convergent validity)

## Methods

The current study was conducted as part of a larger systematic review of smoking cessation trials (for the protocol, see De Bruin et al., 2016; and PROSPERO CRD42015025251). In summary, a search was carried out on November 1st, 2015 using the Cochrane Tobacco Addiction Review Group Specialized Register. Included studies met the following criteria:

1. randomised controlled trials (RCTs) with a minimum follow-up period of 6 months;
2. directed at adult smokers (i.e. 18+ years);
3. trials describing behaviour change interventions, with or without pharmacotherapy, compared to different behaviour change interventions, with or without pharmacotherapy, (b) treatment-as-usual, with or without pharmacotherapy, (c) pharmacotherapy alone, or (d) no treatment;
4. objectively verified (i.e. biochemical verification) smoking cessation rates (primary outcome) reported at least 6 months after the start of the intervention; and
5. published in English in peer-reviewed journals from 1996 onward.

### Materials

First, all published materials for each included study (i.e. articles, protocols, study websites, and other materials in the public domain) were identified. The systematic review team then contacted the authors of all included trials with a request for additional information. Authors were asked to send a) any unpublished materials or descriptions of the support provided to intervention and comparator participants in their trial (e.g. training manuals, self-help materials), and b) to complete a specially developed comparator intervention checklist online. Standardised templates for emails were used for the first contact. If authors did not respond more personalised emailed were sent.

The 76-item checklist (https://osf.io/e834t) consisted of 60 items describing the potential active content of smoking cessation support in the form of activities. Sixteen items assessed other characteristics, namely context (type of provider, mode of delivery, format of delivery, training of the provider, hours of training, tailoring) and treatment time (number of sessions, duration of sessions) of smoking cessation support. The items were descriptions of activities that targeted smoking cessation behaviours (i.e. quitting, abstaining, adhering to medication, engaging in the treatment) and qualified as BCTs. These were derived from smoking cessation guidelines (HSE, 2015; McEwen, 2014; NHS Health Scotland, 2010; Tobacco Use & Dependence Guideline Panel, 2008); a previous study that identified BCTs used in recordings of TAU smoking cessation group sessions (West et al., 2011); a smoking cessation-specific taxonomy of BCTs (Michie et al., 2013; Michie et al., 2011; West et al., 2011); advisory board input (i.e. current or former smokers who previously participated in a smoking cessation programme, smoking cessation professionals, and policy makers); and observations of BCTs delivered in NHS smoking cessation sessions. Study authors were asked to indicate which activities in the checklist were part of the smoking cessation support routinely delivered to the comparator group participants in their trial (i.e. the standard support). Response categories were: ‘Yes’, ‘No’, or ‘I don’t know’. Authors were also asked to report any other relevant comparator intervention activities that were not captured by the checklist.

### Procedure

Corresponding authors of 142 included trials were contacted over a period of 11 months, with a request to send any unpublished materials and to complete the comparator intervention checklist. Six trials did not employ an active comparator and were excluded from the present study. Corresponding authors were contacted several times (as needed), first by email (including 2 reminders), followed up by telephone. If authors did not respond, second and (if non-corresponding) first/last authors were contacted (including two email reminders). If unsuccessful, any remaining authors were contacted by email.

Once all the materials were collected, data were extracted on the content and contextual characteristics. When authors sent existing materials describing their comparator interventions, similar to the items in the checklist, two coders independently identified intervention activities that targeted the four behaviours relevant to smoking cessation (quitting, abstinence, medication management and treatment engagement) and that could be defined as BCTs (Michie et al., 2013; Black et al., 2019). Coders additionally qualified the delivery styles of BCTs (i.e. individual tailoring or active recipient participation; for details, see Black et al., 2019). The following additional contextual characteristics were extracted: provider’s profession, general training of the provider, intervention-specific training of the provider, mode of delivery, and treatment time. When authors sent the comparator checklist, these data were extracted using a syntax. Items reflecting potential active content were considered present when respondents indicated ‘Yes’. When the response was ‘No’ or ‘I don’t know’, BCT linked to the item was considered absent (i.e. not routinely delivered to comparator intervention participants in the trial).

Coders first assessed the primary trial article, then other published materials (such as study protocols), then materials sent by study authors (e.g. training manuals), and finally the checklist. The attributed source of each characteristic was defined as the first instance it was coded in the (1) published article; (2) published supplementary materials; (3) unpublished materials sent by study authors; and (4) the checklist.

### Analyses

Descriptive analyses were used to answer research questions 1 and 2. These analyses described the included trials, response rates and response types for unpublished information (i.e. unpublished materials and/or checklist), and intervention characteristics with their attributed source (i.e. published article, published supplementary materials, unpublished materials sent by study authors, or checklist). Using only studies where authors provided unpublished information, we examined the proportion of the total instances when a comparator intervention characteristic was only identified in unpublished sources.

To answer research question 3, we investigated content validity (representativeness). This was done by examining the extent to which comparator intervention descriptions are representative of the most complete record of content and delivery characteristics obtainable by combining all methods. For representativeness, given the lack of formal criteria, we decided on the threshold of 10% and 20% of the data missing. When more data were missing, we considered the published trial materials as not adequately representing that construct. Specifically, the analyses tested how complete our records are for active content (BCTs) and intervention characteristics (TIDieR components) when (1) only relying on published materials, and (2) when also considering additional data obtained from study authors. This should allow for assessing how much additional information can be obtained when using additional data. The proportion of total instances in which BCTs were only identified in unpublished information sources was also used to descriptively assess whether the representativeness of collecting information on comparator intervention descriptions changes when using combined sources (in comparison to collecting published information only).

To answer research question 4, construct validity of data collection methods was examined. Presumed active content (the construct) was first operationalised as the number of BCTs that were delivered to comparator intervention participants. The known-groups validity and convergent validity of each data collection method was then assessed.

Known-groups validity was assessed by examining the associations between the number of BCTs applied in the comparator intervention, and providers with different behaviour change competency profiles, and different types of comparators. Regarding providers, we would expect counsellors, psychologists, and health educators to deliver more BCTs (i.e. more complex, multi-faceted programmes) than nurses, pharmacists, and physicians (Chapman et al., 2016; Dixon & Johnston, 2010)). Regarding the different types of comparator interventions, we would expect the comparators receiving psychological support to contain more BCTs (Heckman et al., 2010) than brief support; and treatment-as-usual (TAU) groups to be in the middle as this label usually represents a mix of interventions (e.g. Witt et al., 2018).

Convergent validity was assessed by examining the associations between the number of BCTs and treatment time. Because programmes that contain more elements are likely to include, on average, more BCTs and take longer to deliver; and vice versa, programmes that contain more BCTs likely include more elements and thus take longer to deliver, treatment duration was deemed a suitable construct for assessing convergent validity. Again, only the studies for which authors provided unpublished information were included in the analyses.

We also examined convergent validity by examining whether intervention providers with more advanced competency profiles (i.e. counsellor, psychologist or health educator) delivered interventions with more BCTs, than interventions delivered by professionals with less advanced behaviour change competency profiles (i.e. nurses, pharmacists, physicians; Dixon & Johnston, 2010; 2021). Interventions delivered by staff for whom it was not possible to code their competency profile (e.g. ‘research staff delivered the intervention’) were excluded from these analyses. When assessing different types of comparator interventions, the following categories were included: either brief support, TAU or psychological support. When assessing treatment time, studies had to provide information about the comparator treatment time to be included.

To examine the differences in the number of BCTs delivered by different providers, we applied a two-way mixed-model ANOVAs using simple contrasts. We repeated the procedure when examining the differences in the number of BCTs in different control groups using Tamhane’s T2 post-hoc criterion (De Muth, 2014), applied to group comparisons in cases of unequal sample sizes and unequal variances. The dependent variable in both analyses was the number of BCTs, with the within-subject factor consisting of two levels (1: published and 2: combined data collection methods). The between-subject factor consisted of two groups (1: nurses, pharmacists, physicians; 2: counsellors, psychologists and health educators) when assessing providers, and of three groups (1: brief support, 2: TAU, 3: psychological support) when assessing comparator interventions. Šidák’s adjustment for multiple comparisons, similar to Bonferroni’s correction, but controlling for both Type I and Type II errors (Frane, 2015), was applied in both ANOVAs. An interaction effect of data sources (i.e. published versus combined) and provider or comparator type on number of BCTs was computed for both ANOVAs to examine any changes in validity when using combined methods of data collection (compared to collecting published information only). Effect sizes were calculated using partial eta squared (η^2^*_p_*; Lakens, 2013). Eta squared measures the proportion of the variation in Y that is associated with membership of the different groups defined by X. We use η^2^*_p_* to improve comparability of the effect sizes between studies with similar designs (Keppel, 1991).

To examine whether comparator interventions of longer treatment time received more BCTs, a two-level fixed effects model was used nesting BCTs from published and combined sources within studies, with two main effects: source of information (0 = published only; 1 = published and unpublished combined) and treatment time (total number of minutes, centred around the mean), and their interaction effect. With the model set up like this, the fixed effect of treatment time reflects the size of association between the average treatment time and number of BCTs when source = 0, and the interaction effect will reflect the size of the difference in the association when source = 1 compared to source = 0.

All analyses were carried out using SPSS Version 23.

## Results

### Authors’ responses to information requests

Informative responses about comparator interventions were received from 91/136 study authors (66.9%). These comprised 40 (43.9%) who both sent materials and completed the comparator intervention checklist, 12 (13.1%) who only sent materials, and 39 (42.8%) who only completed the checklist.

### Representativeness of the data collection methods

**Figure 1.**
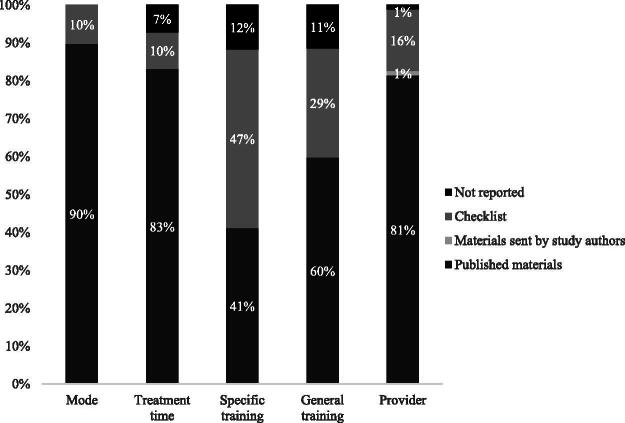
The frequencies of reporting of individual comparator contextual characteristics when examining published and unpublished data sources. Note. Bars omit ‘Materials sent by study authors’ where these did not provide any additional information relevant to that contextual characteristic.

Individual contextual characteristics (Figure 1) could be identified in published sources in 41% (i.e. training of the provider) to 100% (i.e. type of comparator) of the cases. Hence, although individual contextual characteristics are in majority described in the published materials, only mode of delivery was reported more than 90% of the time, and—additionally—general treatment time and provider when an 80% threshold was used. The level of training provided to those delivering the intervention was missing in 40 (general behaviour change or communication training) to 59% (intervention specific training) of cases.

The number of BCTs identified in published and unpublished information sources could be examined for 91 trials for which additional information was obtained from trial authors (see Table 1). The published information sources described only 26% of total coded BCTs. When authors provided both unpublished comparator materials and completed a checklist (*N* = 40), an additional 33.9% of information was found in the materials; and an additional 44.6% of total BCTs were only identified through the checklist (see Table 1). It is important to remember that does not necessarily represent all the BCTs that could be obtained through additional data sources, as the coders extracted only the BCTs that have previously not appeared in any of the materials. This means, for example, that the same BCTs might be found in unpublished materials and the checklist. However, the source of information for that BCT would be noted as unpublished materials, because that was the origin of its first mention. These results indicate that with about three-quarters of the data missing (substantially more than the 10-20% missingness thresholds we apply here), published intervention materials do not qualify as a representative data source for interventions’ presumed active content.

**Table 1.** Sources of comparator intervention BCTs in smoking cessation trials that provided a completed checklist and/or unpublished materials.

	*N* of BCTs	% of total BCTs
Source of information (N = 91)*		
Published article	304	16.2
Published supplementary materials	173	9.2
Unpublished materials sent by authors	413	21.9
Checklist	986	52.6
Total	1882	100.0
Source of information (N = 40)**		
Published article	154	15.1
Published supplementary materials	64	6.4
Unpublished materials sent by authors	334	33.9
Checklist	454	44.6
Total	1017	100

*Note.* *For 91 studies, authors provided additional information either in the form of unpublished materials, checklist or both. **For 40 studies, authors provided additional information both in the form of unpublished materials and the checklist.

### Known-groups and convergent validity of comparator intervention descriptions

To examine the construct validity of used methods, we assessed whether there were differences in the number of BCTs (identified in published and combined information sources) provided by different types of providers (research question 4a) and comparators (research question 4 b), and for different treatment time (research question 4c). Descriptive statistics for different types of providers and comparators are shown in Table 2. We assessed the differences in all studies that provided additional information (either via the checklist or unpublished materials).

**Table 2.** Number of BCTs identified in published and combined information sources for different types of providers and different types of comparator interventions.

		Published		Combined	
	*N*	M	SD	M	SD
Provider	73				
Nurses, physicians, pharmacists	28	4.28	5.5	13.35	12.43
Psychologists, counsellors	45	6.16	5.47	26.98	14.48
Type of comparator intervention	84				
Brief support	24	5.42	5.98	13.17	11.36
Treatment-as-usual	40	4.45	5.02	19.88	15.26
Psychological support	20	7.90	6.34	35.05	8.92

*Note.* Provider information available for 73 of 91 trials (80.2%); Information on type of comparator intervention available for 84 of 91 trials (92.3%).

The results of the ANOVAs that assessed known-group validity by examining the number of BCTs provided by different types of providers (4a) and comparators (4 b) are shown in Table 3. The interaction effect, significant in both ANOVAs, and simple effects indicate significant differences in the number of BCTs provided by different providers and in different types of comparators, but only when additional information was obtained from study authors.

**Table 3.** Simple and interaction effects results found for the differences in the N of BCTs for different types of provider (research question 4a) and types of comparator (research question 4 b) in published and combined information sources.

Effect	MS	Df	*F-*statistic	*p* value*	η^2^*_p_*
Provider (published)	62.31	1	2.07	0.15	
Provider (combined)	3277.62	1	17.42	< 0.01	
Provider x Source	1218.05	1	16.42	< 0.01	0.18
Type of comparator (published)	79.64	2	2.51	0.09	
Type of comparator (combined)	2721.96	2	16.25	< 0.01	
Type of comparator x Source	1033.28	2	15.91	< 0.01	0.28

Note. Provider information available for 73 of 91 trials (80.2%); information on type of comparator intervention available for 84 of 91 trials (92.3%).

Average reported offered treatment time was 156.46 minutes (SD = 264.82) in eligible comparators for which authors provided additional information. An interaction effect with information source (γ = 0.020, p < .01, 95%CI [0.009; 0.030]) indicated that there was a stronger association between treatment time and number of BCTs when extracting the information from combined sources (γ = 0.017, p < .01, 95%CI [0.008; 0.028]) than from published alone (γ = −0.003, p = .129, 95%CI [-0.006; 0.001]). Hence, these results indicated both known-groups validity and convergent validity of data collection methods when using all data sources, but not when using only the published information.

## Discussion

The present study examined the feasibility and validity of two methodological approaches to collecting unpublished information on comparator interventions in systematic reviews of behavioural trials. The individual contextual characteristics of comparator interventions were reported well in the public domain, apart from the information on the training of the provider, which was improved with the use of the checklist. The active content of comparator interventions was reported poorly in the published sources. The representativeness of the reported active content was improved through collecting additional information, the majority of which was captured through the checklist. The use of combined data sources appears to be more representative of the full intervention descriptions than relying on published materials only. In addition, using the combined approach led to higher known-groups and convergent validity of the results as an indicator of active content. This suggests that collecting additional information increases the confidence in conclusions regarding presumed active content of interventions being drawn from systematic reviews (de Bruin et al., 2021; de Bruin et al., 2010).

Collecting information about comparator interventions through a pre-designed checklist that is based on research, evidence-based practice, and input from various stakeholders had a higher success rate (more authors providing information via checklist and more comprehensive reporting of the active content) than collecting information through researchers coding unpublished materials. More authors supplied information when provided with the checklist: even when unpublished materials were available, the checklist provided additional information on active content. A possible explanation is that unpublished materials do not capture the whole range of support offered in comparator interventions. For example, TAU might consist of a leaflet and brief counselling from a nurse, but the content of that counselling is unlikely to be formally described in a written protocol (Byrd-Bredbenner et al., 2017; Freedland et al., 2011), thus not being captured when collecting the materials on comparators. Using a checklist with a list of potential activities can serve as a prompt to remind authors of the additional active content in their comparator interventions. One may wonder whether using a checklist may lead to overreporting of active content due to social desirability. However, given that there is no evident motive for authors to overreport comparators, and since the active content scale shows high internal consistency reliability and predictive validity both in a prior meta-analysis of HIV medication adherence controls and in the current smoking cessation review (Black et al., 2020; de Bruin et al., 2009), we think this is unlikely.

The checklist represents an efficient (and feasible to use) instrument for systematic reviewers. However, systematic reviewers should take into consideration the time essential to develop instruments that capture the activities offered to comparator interventions in behavioural trials and the time donated by the study authors to complete the checklist. Use of different national guidelines (e.g. Tobacco Use & Dependence Guideline Panel, 2008), and coded observation of practice should be considered when developing this type of checklist, as well as the use of advisory boards and various stakeholders.

The distribution of the information sources (published and unpublished) of active content in our study is in line with previous findings (Black et al., 2020; Byrd-Bredbenner et al., 2017) that comparator interventions are poorly reported in the public domain. Published sources in our study offered only 26% of the total information on presumed active content in studies where authors provided additional information. When authors provided both unpublished materials and the checklist, the checklist was found to be the most comprehensive tool for collecting the information on active content, containing more than half of the intervention BCTs not identified in other materials. The checklist collected 47% of the reported information regarding the intervention-specific training of the comparator intervention provider and 70.6% of the information regarding their general training. Other contextual characteristics, such as type of the comparator (100%), treatment time of the comparator intervention (91.8%), and provider of the comparator intervention (83.6%) appear to be relatively comprehensively described in the published descriptions of smoking cessation trials, which might indicate a limited need for additional data collection methods for these characteristics. This matches with previous research showing these variables to be reasonably well reported in publications (Byrd-Bredbenner et al., 2017). Both presumed active content and contextual characteristics can determine outcomes of comparator interventions (Bishop et al., 2015). As such, comprehensive reporting of the contextual characteristics means published materials can be of utility when extracting the information on comparator groups. It is worth noting that these characteristics might be more easily or comprehensively collected with the use of a checklist.

The present study aimed to examine different facets of validity of collecting additional information. Representativeness of the comparator intervention description as the most complete record of content and contextual characteristics was found when combining all methods for active content (number of BCTs). Published materials were found to be a representative record of the examined contextual characteristics, apart from the intervention-specific training of the provider, where combining published materials and the checklist offered the most complete record of this characteristic. These results show that the information on comparator intervention active content and some contextual characteristics are not representative of the full intervention description when using published materials only.

We also assessed known-groups and convergent validity of the results when extracting the information from published and additional data sources. We hypothesised that more highly-qualified, trained providers (known-groups validity) would be associated with a higher number of BCTs (the presumed active content) in more complex interventions (known-groups validity), and in longer treatments (convergent validity). The expected patterns (healthcare providers delivering fewer BCTs than counsellors; psychological support consisting of more BCTs than TAU or brief support comparator interventions; positive associations between treatment time and the number of BCTs) were only seen when examining information from combined published and unpublished sources. This was further confirmed by significant interactions between the information sources and intervention properties and post-hoc examinations. These results of three different assessments of the operationalised construct (number of BCTs) indicate that the validity of the published information on the presumed active content should not be assumed. Employing additional data collection methods can improve the known groups and convergent validity of the results as an index of active content. However, it is currently not possible to determine whether an individual additional data collection approach (unpublished materials vs. checklist) is superior to the other (see Limitations and future directions).

### Limitations and future directions

The present study has limitations related to its sample size and design. The sample size was solely determined by the number of qualifying studies within the systematic review at the time of extraction (2016). This affects the interpretation of findings. Specifically, the conclusions regarding validity of the present study relate to the use of combined unpublished (i.e. unpublished materials and the checklist) information sources in addition to published materials. Because of the number of eligible studies in this review, we were unable to identify improvements in the validity of data that could be specifically attributed to unpublished materials or the checklist. Revisiting this question in a larger pool of eligible trials may be of useful, especially for reviewers who wish to know the most efficient means of obtaining valid and reliable data on comparator interventions. While the checklist appears to improve representativeness, analyses focusing solely on the checklist’s validity are beyond the scope of this study. To determine the real benefit of the use of either approach (i.e. retrieving unpublished materials or checklist), validity analyses should be carried out on a larger sample of studies. These studies should compare all information, collected in the public domain, with the information, collected from unpublished materials, and the checklist information.

The design of this systematic review coding means we cannot determine which of the data sources provided the most information on its own. BCTs in this study were coded in the first instance in the materials and not subsequently. As such, the information on whether there the BCTs are reported in both published materials and those, sent by study authors, is not available. This means that in this study, there is no information available on which BCTs were found in both published materials and materials sent by study authors. We could identify the total number of BCTs, identified through the checklist, and assess which were found in both published materials and the checklist. However, because this is not possible to do for materials, sent by study authors, this study does not provide a complete picture on all BCTs in individual additional data sources. However, future studies investigating additional data collection methods should consider coding the complete active content in all data sources and comparing the representativeness of reporting between them.

We operationalised the active content as the number of BCTs in the intervention. This assumed that all BCTs is equally important, which is unlikely to be the case. Individual BCTs could be weighted based on their effectiveness in other studies, but such information was not available at the time of writing.

Small effect sizes were found for the interaction between information sources and different intervention properties. A combined data collection approach might contribute to improved understanding of comparator interventions, but its effect on the expected differences is small. Further examination of collecting additional information through unpublished materials and a checklist in other domains of health behaviour trials is needed to see whether the findings of this study generalizes to other contexts.

Finally, we did not collect fine-grained data on the number of reminders we had to send authors and the timing of their response. In future studies, it would be valuable to systematically collect data on the number/percent of studies that required further follow-up via additional emails/telephone calls, the number/percent of studies in which additional authors (besides the corresponding author) were contacted, the mean number of contact attempts needed per study, and the average duration (e.g. number of days) it took for study authors to provide the requested information. This would be useful for deciding on the feasibility and planning of additional data collection in future systematic reviews.

## Conclusions

The implications arising from poor reporting of comparators can affect (i) replicability of the trials (ii) the possibility of successful implementation of the intervention into a different setting, and (iii) our understanding of variability between comparators (de Bruin et al., 2021; Chalmers & Glasziou, 2009; Boutron et al., 2008; Wagner & Kanouse, 2003). The present study shows that comprehensive information on the content of comparators can be successfully and validly extracted through collecting unpublished materials and through a purpose-built checklist. A checklist, developed for the purpose of a systematic review, appears to be more feasible and comprehensive than collecting unpublished information in the form of (usually) written materials from study authors. Systematic reviewers should consider adopting a similar methodological approach when conducting reviews of behavioural interventions—not only for comparator, but also for intervention treatments (for details, see Black et al., 2020).

## Supplementary Material

Supplemental Material
