# Supplementary material for: Pragmatic evaluation of methods for retrieving unpublished information on comparator interventions in a systematic review of smoking cessation trials
Source: Psychol Health. 2022 Jul 23;39(4):435–51. doi: 10.1080/08870446.2022.2081688 (PMC10911680; doi:10.1080/08870446.2022.2081688)
Supplement: Supplemental Material [file GPSH_A_2081688_SM1141.docx]

Appendix VIII. Specially-developed comparator group checklist, sent to study authors in a systematic review of behavioural smoking cessation trials

| \| IC-SMOKE: Control group support questionnaire \| \| --- \|  \|  \| \| --- \|  \|  \| We are undertaking a systematic review to identify the effective behavioural components of smoking cessation interventions evaluated in randomised controlled trials published after 1995. This research is funded by Cancer Research UK. Our aim is to examine, in detail, the active content of the smoking cessation support provided to intervention and control group participants. \| \| --- \| --- \|  \|  \| \| --- \|  \|  \| Control or comparison groups in smoking cessation trials often receive some form of support focused on smoking cessation (‘active controls’). Control group participants may receive ‘treatment-as-usual’, typically the smoking cessation support provided by existing smoking cessation services. Other control groups might (additionally) receive standardised support introduced by the research team, for instance ‘brief advice to quit smoking’ or ‘self-help materials’. \| \| --- \| --- \|  \|  \| \| --- \|  \|  \| We want to know what support the control group participants in smoking cessation trials received. We developed a questionnaire that allows study authors to report this information. The first part asks for general characteristics of control group support. The second part contains detailed questions about the potential active content of the control group support. Free text boxes allow you to report any additional relevant characteristics of control support not covered by the questionnaire. The questionnaire should take 10-15 minutes to complete. \| \| --- \| --- \|  \|  \| \| --- \|  \|  \| Thank you in advance for your help. Completion of this questionnaire is of crucial importance for this project and should yield unique data that will help us to better interpret smoking cessation trials. \| \| --- \| --- \|  \|  \| \| --- \|  \|  \| On behalf of the research team, \| \| --- \| --- \|  \|  \| \| --- \|  \|  \| Maarten Eisma, PhD (researcher) Marijn de Bruin, PhD (principal investigator) \| \| --- \| --- \|  \|  \| \| --- \|  \|  \| Contact details in case of any questions or comments: Email: stop_smoking_review@abdn.ac.uk \| \| --- \| --- \| |  |
| --- | --- | --- | --- | --- | --- | --- | --- | --- | --- | --- | --- | --- | --- | --- | --- | --- | --- | --- | --- | --- | --- | --- | --- |
|  |  |

| \| Study Identification \| \| --- \|  \|  \| \| --- \|  \| **Could you please provide the following information about the article mentioned in our email?** \| \| --- \|  \|  \| \| --- \|      \|  \| First author name: \| \| --- \| --- \| \|  \|  \|  \| \| --- \|      \|  \| Your name (if different from the first author): \| \| --- \| --- \| \|  \|  \|  \| \| --- \|      \|  \| Study Identifier (First author + Publication year, e.g. Smith2016): \| \| --- \| --- \| \|  \|  \|  \| \| --- \|  \| Part 1. General information about control group support \| \| --- \|  \|  \| \| --- \|  \| **The following questions relate to the general characteristics of the smoking cessation support provided to the control group participants in your trial.** \| \| --- \|  \|  \| \| --- \|      \|  \| **1. Did your control group receive treatment-as-usual, support developed by the researchers for the purpose of this trial, or a combination?** \| \| --- \| --- \| \| \|  \| The control group recieved treatment-as-usual \| \| --- \| --- \| \| \| \|  \| The control group received support developed by the research team \| \| --- \| --- \| \| \| \|  \| The control group received both treatment-as-usual and support developed by the researchers \| \| --- \| --- \| \| \| \|  \| The control group support was designed differently, namely: \| \| --- \| --- \| \|  \|  \|  \| \| --- \| --- \| \|  \|  \|  \| \| --- \|      \|  \| **2. What were the reasons for selecting the control group support delivered in your trial?** \| \| --- \| --- \| \|  \|  \|  \| \| --- \|      \|  \| **3. In some trials, intervention groups receive the smoking cessation intervention *plus* the support that the control group participants receive. In other trials, they only receive the intervention and not any of the control group support. How was that in your trial?** \| \| --- \| --- \| \| \|  \| The intervention group(s) only received the smoking cessation intervention \| \| --- \| --- \| \| \| \|  \| The intervention group(s) received the control group support *plus* the smoking cessation intervention \| \| --- \| --- \| \| \| \|  \| The trial was designed differently, namely: \| \| --- \| --- \| \|  \|  \|  \| \| --- \| --- \| \|  \|  \|  \| \| --- \|      \|  \| **4.** **The professional(s) who delivered the control group support (including any written material) was a:** *Check all that apply* \| \| --- \| --- \| \| \|  \| Primary care nurse (outpatient setting) \| \| --- \| --- \| \| \| \|  \| Secondary care nurse (inpatient setting) \| \| --- \| --- \| \| \| \|  \| Physician \| \| --- \| --- \| \| \| \|  \| Pharmacist \| \| --- \| --- \| \| \| \|  \| Smoking cessation counsellor \| \| --- \| --- \| \| \| \|  \| General practitioner \| \| --- \| --- \| \| \| \|  \| Researcher or research assistant \| \| --- \| --- \| \| \| \|  \| Other, namely: \| \| --- \| --- \| \|  \|  \|  \| \| --- \| --- \| \|  \|  \|  \| \| --- \|  \| This person (these persons) are from now on referred to as the *healthcare provider(s)* in the remainder of this questionnaire \| \| --- \|  \|  \| \| --- \|      \|  \| **5. Was the health care provider trained in general patient education or behaviour change counselling strategies?** *(e.g., motivational interviewing)* \| \| --- \| --- \| \| \|  \| Yes, extensively trained \| \| --- \| --- \| \| \| \|  \| Yes, received some training \| \| --- \| --- \| \| \| \|  \| No, received (almost) no training \| \| --- \| --- \| \| \| \|  \| I don’t know \| \| --- \| --- \| \| \| \|  \| Not applicable (support was not delivered in person) \| \| --- \| --- \| \|  \|  \| \| --- \|      \|  \| **6. Was the health care provider trained specifically for delivering the smoking cessation support provided in the control group?** \| \| --- \| --- \| \| \|  \| Yes, received extensive training \| \| --- \| --- \| \| \| \|  \| Yes, received some training \| \| --- \| --- \| \| \| \|  \| No, received (almost) no specific smoking cessation training \| \| --- \| --- \| \| \| \|  \| I don’t know \| \| --- \| --- \| \| \| \|  \| Not applicable (support was not delivered in person) \| \| --- \| --- \| \|  \|  \| \| --- \|      \|  \| **7. How many hours of training did the health care providers receive specifically for delivering the smoking cessation support provided in the trial?** *If no training was provided, please fill out '0'* \| \| --- \| --- \| \|  \|  \|  \| \| --- \|      \|  \| **8. In what mode(s) was control group support delivered?** *Check all that apply* \| \| --- \| --- \| \| \|  \| Face-to-face: Individual \| \| --- \| --- \| \| \| \|  \| Face-to-face: Group \| \| --- \| --- \| \| \| \|  \| Telephone \| \| --- \| --- \| \| \| \|  \| E-mail \| \| --- \| --- \| \| \| \|  \| Text-messaging \| \| --- \| --- \| \| \| \|  \| Website \| \| --- \| --- \| \| \| \|  \| Written (paper-based) material \| \| --- \| --- \| \| \| \|  \| Other mode of delivery, namely: \| \| --- \| --- \| \|  \|  \|  \| \| --- \| --- \| \|  \|  \|  \| \| --- \|      \|  \| **9. In what setting was control group support delivered?** *This may not be applicable if control group support was not delivered in person. If so, please fill out 'not applicable'* \| \| --- \| --- \| \|  \|  \|  \| \| --- \|      \|  \| **10. In how many sessions or other units of exposure (e.g. email messages) was control group support delivered?** *This may not be applicable if control group support was not delivered in person. If so, please fill out 'not applicable'* \| \| --- \| --- \| \|  \|  \|  \| \| --- \|      \|  \| **11. How much time did the delivery of control group support take in total?** *This may not be applicable if control group support was not delivered in person. If so, please fill out 'not applicable'* \| \| --- \| --- \| \|  \|  \|  \| \| --- \|      \|  \| **12. Was the *content* of the control group support tailored to characteristics of the client?** \| \| --- \| --- \| \| \|  \| No, control group support content was not tailored to individual characteristics \| \| --- \| --- \| \| \| \|  \| Yes, some of the control group support was tailored to individual characteristics \| \| --- \| --- \| \| \| \|  \| Yes, all/most of the control group support was tailored to individual characteristics \| \| --- \| --- \| \|  \|  \| \| --- \|      \|  \| **13. Was the *amount* of the control group support tailored to the needs of the client?** \| \| --- \| --- \| \| \|  \| No, all clients received the same amount of smoking cessation support \| \| --- \| --- \| \| \| \|  \| Yes, clients who required more smoking cessation support were offered more support \| \| --- \| --- \| \|  \|  \| \| --- \|      \|  \| **14. Were control group participants offered stop smoking medication? *(****e.g. Nicotine Replacement Therapy products, varenicline, bupropion)* \| \| --- \| --- \| \| \|  \| No \| \| --- \| --- \| \| \| \|  \| Yes, namely … \| \| --- \| --- \| \|  \|  \|  \| \| --- \| --- \| \|  \|  \|  \| \| --- \|      \|  \| **15. For interventions, researchers often assess how many of the intervention participants were exposed to how much of the intervention. Do you know how many of the control group participants were exposed to how much of the control group support?** \| \| --- \| --- \| \| \|  \| No \| \| --- \| --- \| \| \| \|  \| Yes, namely … \| \| --- \| --- \| \|  \|  \|  \| \| --- \| --- \| \|  \|  \|  \| \| --- \|      \|  \| **16. Please provide any additional comments if you feel that the above questions have not adequately captured the relevant characteristics of your control group support** *(note that questions on the support content will appear in Part 2 of the questionnaire)* \| \| --- \| --- \| \|  \| |  |
| --- | --- | --- | --- | --- | --- | --- | --- | --- | --- | --- | --- | --- | --- | --- | --- | --- | --- | --- | --- | --- | --- | --- | --- | --- | --- | --- | --- | --- | --- | --- | --- | --- | --- | --- | --- | --- | --- | --- | --- | --- | --- | --- | --- | --- | --- | --- | --- | --- | --- | --- | --- | --- | --- | --- | --- | --- | --- | --- | --- | --- | --- | --- | --- | --- | --- | --- | --- | --- | --- | --- | --- | --- | --- | --- | --- | --- | --- | --- | --- | --- | --- | --- | --- | --- | --- | --- | --- | --- | --- | --- | --- | --- | --- | --- | --- | --- | --- | --- | --- | --- | --- | --- | --- | --- | --- | --- | --- | --- | --- | --- | --- | --- | --- | --- | --- | --- | --- | --- | --- | --- | --- | --- | --- | --- | --- | --- | --- | --- | --- | --- | --- | --- | --- | --- | --- | --- | --- | --- | --- | --- | --- | --- | --- | --- | --- | --- | --- | --- | --- | --- | --- | --- | --- | --- | --- | --- | --- | --- | --- | --- | --- | --- | --- | --- | --- | --- | --- | --- | --- | --- | --- | --- | --- | --- | --- | --- | --- | --- | --- | --- | --- | --- | --- | --- | --- | --- | --- | --- | --- | --- | --- | --- | --- | --- | --- | --- | --- | --- | --- | --- | --- | --- | --- | --- | --- | --- | --- | --- | --- | --- | --- | --- | --- | --- | --- | --- | --- | --- | --- | --- |
|  | |

Part 2: The content of the control group smoking cessation

**You will now be presented with a list of items designed to capture the active content of the smoking cessation support provided to control groups. Some of these items are very common, others may be rarely presented to control groups. We would like to know which of these items the *majority of the control group participants* in your trial were exposed to. Hence, we want to capture the items that belong to the ‘*standard smoking cessation support’* provided, rather than items delivered occasionally to a subgroup of clients.**

**Please indicate for each item whether it was part of the standard smoking cessation support for your control group by selecting ‘Yes' ‘No’ or ‘I don’t know’.**

The majority of the control group participants in my trial was supported to stop smoking through…

1. ...providing information about the health consequences of smoking
2. ...providing information about the social and environmental consequences of smoking (for example, inform the client how much money smoking costs)
3. ...providing information about the emotional consequences of smoking (e.g., informing the client that smoking cessation increases happiness and life-satisfaction)
4. ...providing information about the health consequences of second-hand smoke for others
5. ...emphasizing the negative health consequences of smoking (e.g., by showing cigarette packs with pictures of diseased lungs)
6. ...advising the client to stop smoking
7. ...encouraging the client to stop smoking
8. ...encouraging the client to identify and compare advantages and disadvantages of smoking cessation
9. ...asking the client to describe any previous success in giving up smoking (e.g., by asking how long the client managed to stay quit before)
10. ...suggesting the client deliberately adopts a different, more positive view on past failed quit attempts or lapses (e.g., by suggesting that past lapses can be viewed as learning experiences that can help with this quit attempt)
11. ...advising the client to engage in more smoking than usual (e.g., smoking twice as much as normal) to reduce the motivation to smoke
12. ...explaining to the client that they would make a good role-model to others (e.g., their children) if they quit smoking
13. ...drawing attention to inconsistencies between the clients’ smoking behaviour and the clients’ preferences, values or goals
14. ...motivational interviewing
15. ...advising on, or arranging social support by family members, friends, or colleagues to facilitate smoking cessation
16. ...telling the client how to use a Carbon Monoxide (CO) monitoring device, measure CO-levels, and give feedback on measured CO-levels
17. ...referring patients to another information source or service, such as a stop smoking website or telephone helpline

Other activities to inform and motivate clients to quit smoking were delivered, namely:

|  |
| --- |

The following items are about *supporting clients in planning to quit smoking.* Please indicate for each item whether it was part of the *standard smoking cessation support* for your control group by selecting one of the response options below.

The majority of the control group participants in my trial was supported to stop smoking through…

1. ...agreeing a quit date with the client
2. ...instructing the client how to stop smoking (e.g., stress the importance of abrupt cessation rather than cutting down)
3. ...analysing barriers to quit smoking and selecting effective strategies to overcome these barriers
4. ...analysing triggers to smoke with the client and coming up with or selecting effective ways of coping with these triggers
5. ...advising the client to remove all tobacco products, lighters and ashtrays from his/her surroundings
6. ...advising the client to ask smoking family members, housemates, friends and/or colleagues not to smoke in their presence and not to leave their cigarettes in view
7. ...agreeing with the client that they will engage in other neutral or less harmful behaviour when feeling the urge to smoke (e.g., engaging in brief exercise)
8. ...encouraging the client to set small targets (e.g., get through a morning without smoking), making them increasingly difficult (e.g., get through a whole day without smoking) until one has achieved a larger target (e.g., get through a week without smoking)
9. ...encouraging the client to restrict their smoking to certain locations before the first quit date
10. ...advising the client to avoid situations in which common triggers to smoking occur (e.g., by changing daily/weekly routines)
11. ...advising the client to distract themselves when feeling the urge to smoke
12. ...advising on ways of dealing with withdrawal symptoms
13. ...advising to engage in positive self-talk in situations where the client experiences strong urges to smoke

Other activities to support clients in planning smoking cessation were delivered, namely:

|  |
| --- |

The following items are about *supporting abstinence* after client have quit smoking. Please indicate for each item whether it was part of the *standard smoking cessation support* for your control group by selecting one of the response options below.

The majority of the control group participants in my trial was supported to abstain from smoking through…

1. ...encouraging or praising the client for staying abstinent
2. ...encouraging or praising the client who has slipped, for the effort made to try to stay abstinent
3. ...asking the client to assess how much regret they will feel if they start smoking again
4. ...advising the client to save the money not spent on smoking and use the money on something they value (e.g., a fun activity)
5. ...informing the client that money, vouchers or valued objects will be given to them if they have stayed abstinent for a certain amount of time
6. ...arranging for the client to receive money, vouchers or valued objects if they have stayed abstinent for a certain amount of time
7. ...arranging for the withdrawal of something valued if the client lapses (e.g., subtract money from a refundable deposit when a cigarette is smoked)
8. ...arranging for rewards to be given to the client only if they keep extending the time they do not smoke (e.g., first reward given after a day, second reward after a week, third reward after a month)
9. ...analysing barriers to stay abstinent with the client and come up with or select effective strategies to overcome these barriers
10. ...asking the client to perform behaviours to reduce negative emotions, such as relaxation exercises, to make it easier to stay abstinent
11. ...asking the client to formulate a new identity as a “non-smoker”
12. ...explaining to a client how to stay abstinent (e.g., stress the importance of not smoking “a single puff” after the quit date)
13. ...advising the client to make a plan to deal with strong urges to smoke when these occur in the future, after the treatment
14. ...asking the client to use an “I will” statement to affirm or reaffirm a strong commitment (e.g., using the words “strongly”, “committed” or “high priority”) to stay abstinent
15. ...providing medication or referring the client to a specialist when they experience strong negative mood or depression after the quit date

Other activities to support clients in maintaining abstinence were delivered, namely:

|  |
| --- |

The following items are about *correctly using stop smoking medication*. Please indicate for each item whether it was part of the *standard smoking cessation support* for your control group by selecting one of the response options below.

The majority of the control group participants in my trial was supported in correctly using stop smoking medication through…

1. ...providing stop smoking medication or enacting necessary procedures for clients to obtain stop smoking medication
2. ...advising the client how to use stop smoking medication effectively
3. ...making a plan with the client about which stop smoking medication should be used, how to get a supply and/or when to start using it
4. ...asking the person to use an “I will” statement to affirm or reaffirm a strong commitment (e.g., using the words “strongly”, “committed” or “high priority”) to use stop smoking medication as prescribed.
5. ...encouraging the client to ask a family member, friend and/or housemate to help obtain, or remember to take, stop smoking medication
6. ...introducing objects or cues that will remind the person to take their stop smoking medication (e.g., a bathroom mirror sticker, a text message)
7. ...gradually reducing the number of cues or reminders a client receives to take their stop smoking medication
8. ...arranging for the removal of something unpleasant (e.g., household chores) if the client adheres to stop smoking medication for a specified period of time

Other activities to support the clients’ medication management were delivered, namely:

|  |
| --- |

The following items are about *engaging clients in the smoking cessation programme*. Please indicate for each item whether it was part of the *standard smoking cessation support* for your control group by selecting one of the response options below.

Engagement of the majority of control group participants in my trial was supported through…

1. ...providing information about the advantages of engaging in the treatment programme (for example, proven effectiveness)
2. ...agreeing with the client to participate in relevant parts of the smoking cessation programme
3. ...arranging for a client to receive money, vouchers or valued objects if they participate in in relevant parts of the stop smoking programme

Other activities to support the client to engage in the stop smoking programme were delivered, namely:

|  |
| --- |

The following items are about *group support.* Please indicate for each item whether it was part of the *standard smoking cessation support* for your control group by selecting one of the response options below

The majority of the control group participants in my trial was supported to stop smoking in a group by …

1. ...organising a “betting game” (e.g., buddies each bet an agreed amount of money on not smoking during the next week. If one buddy smokes, the other gets the money. If both smoke, the money is given to an agreed charity)
2. ...inviting group members to compare their CO-readings with each other
3. ...organising a group discussions about smoking and quitting, in which clients are encouraged to compare experiences, with a focus on what coping strategies may be effective during their next quit attempt
4. ...suggesting that, when formal treatment is over, the group meet at a mutually convenient location and time to continue supporting each other

Other group activities were delivered to support the client to stop smoking, namely:

|  |
| --- |
